# Effect of short-stay service use on stay-at-home duration for elderly with certified care needs: Analysis of long-term care insurance claims data in Japan

**DOI:** 10.1371/journal.pone.0203112

**Published:** 2018-08-29

**Authors:** Yoko Moriyama, Nanako Tamiya, Akira Kawamura, Thomas D. Mayers, Haruko Noguchi, Hideto Takahashi

**Affiliations:** 1 Department of Health and Welfare Services, National Institute of Public Health, Saitama, Japan; 2 Department of Health Services Research, Faculty of Medicine, University of Tsukuba, Ibaraki, Japan; 3 Health Services Research & Development Center, University of Tsukuba, Ibaraki, Japan; 4 School of Political Science and Economics, Waseda University, Tokyo, Japan; 5 Medical English Communications Center, Faculty of Medicine, University of Tsukuba, Ibaraki, Japan; 6 National Institute of Public Health, Saitama, Japan; National Yang-Ming University, TAIWAN

## Abstract

**Objective:**

Home independence is an important issue for the elderly in many countries and cultures. The aim of this study was to examine the effect of short-stay service use on stay-at-home duration for elderly people by level of care need under the Japanese long-term care insurance system.

**Methods:**

We analyzed anonymous, Ministry of Health, Labour and Welfare of Japan Long-Term Care Insurance claims data from Ibaraki Prefecture. All participants were certified as eligible for long-term care insurance and had moved into a facility under long-term care insurance after certification between April 2006 and March 2012. Data was analyzed for 2,454 participants aged 65 years or older who entered residential care at least 1 month after initial use of care services. The participants were divided into 2 groups (low- and high-care need), depending on their required level of care. Cox proportional hazard modeling was used to calculate the adjusted hazard ratio (HR) of residential care admission after initial use of care services.

**Results:**

Use of short-stay services was positively correlated to delay of residential care admission compared to non-use in the low-care need group (HR; 0.834, 95% confidence interval (CI); 0.740–0.939). In the high-care need group, however, use of short-stay services was somewhat correlated with earlier admission (HR; 1.254, 95% CI; 1.084–1.451).

**Conclusions:**

The results of this study show that appropriate timing short-stay service use is necessary for the elderly to stay at home longer.

## Introduction

Faced with a rapidly aging society, Japan, like many countries of the world, has found it imperative to develop a sustainable long-term care system. To this end, the Japanese government initiated a mandatory, public, long-term care insurance (LTCI) system in 2000. The LTCI system is designed to allow elderly people to live an independent daily life in their home in accordance with the limits of their physical condition [1]. Under the current system, individuals who desire access LTCI services must apply to the local government where they are certified or rejected according to age and physical and mental condition, but with no consideration of income and family situation [2]. The applicant receives a certification of need, which is categorized into 7 levels: support levels 1 and 2 and care need levels 1 to 5. Support levels 1 and 2 indicate that the person does not need formal care, but his or her condition requires some kind of assistance with daily living or preventive care. Care need levels 1 to 5 indicate that the person requires formal care, with care level 1 signaling the least severe and level 5 the most severe level of care need [3]. Certified persons are eligible to receive services under the LTCI.

As in other developed countries, home-based care is promoted in Japan, being preferential for a number of reasons: firstly, the long waiting lists for nursing home admission [4]; secondly, nursing home care is more expensive than remaining in the community [5]; thirdly, many elderly people prefer to receive care at their own home rather than going into a nursing home; and, finally, family caregivers also tend to prefer home care for their elderly relatives [6].

Home care by family caregivers plays a vital role in supporting the disabled elderly. In Japan, women in particular have taken on heavy responsibilities as caregivers. Some studies have brought to light the various burdens of caregiving, namely: stress, poor psychological and physical health, and low levels of well-being [7–9]. As a means to support family caregivers, the LTCI promoted the socialization of elderly care under the slogan “from care by family to care by society.” Previous studies of the effects of LTCI service use on caregiver burdens have reported mixed results, but the underlying point is that there is still not enough support available [10–12]. At the time of this study, Japan actually has no legal system of direct support for caregivers with the exception of its introduction of care leave. However, while LTCI services are targeted primarily at care recipients, respite care is partially aimed at family caregivers to give them “respite” from the demands of caregiving. Short-stay service under the LTCI, consisting of a few nights at a time in residential care, is one form of respite care offered in Japan. Additionally, some surveys of caregivers in Japan have revealed that the support that is in highest demand is a short-stay service with no appointment, much like an emergency care service [13,14]. Furthermore, caregivers believe that the availability of such a service (being assured of short-stay service at any time) would help them to continue caring for their elderly family member [14,15]. In this current study, therefore, we focused upon the provision of short-stay service. We hypothesized that the improvement of short-stay services and subsequent usage of such services, would lead to an increase in the duration by which the elderly can stay at home.

Some studies have described the impact of short-stay service use on outcome-based measures such as changes in the level of care need, psychological and physical conditions of elderly, and caregiver burden [16–24]. On the other hand, after the implementation of LTCI in Japan, very few studies have reported about the impact of short-stay service use on stay-at-home duration. Tomita and colleagues reported that those elderly who used short-stay services were less likely than non-users to be hospitalized or enter residential care. They measured the duration from initial certification of eligibility for LTCI benefits and performed an analysis of all participants and a sub analysis of those of low-care need levels [25]. On the other hand, Ishizuki and colleagues performed a case-control study among elderly in the high-care need levels (certified as care levels 4 and 5) and found that amongst elderly nursing home residents, those who had recently entered after living in their homes had more frequent use of short-stay services in comparison to long-term dwellers of 3 years or more [26].

Measuring the stay-at-home duration from initial certification of eligibility for LTCI benefits, as in the study by Tomita and colleagues, might not be the best point of measurement. One study suggests that about 20% of certified elderly do not use services [27] and of the participants in that study, about 55% did not begin using care services immediately following their certification. This is important because during the interval between certification and service use the care level may have changed. Many non-users among the support levels and low-care need levels have described that they applied for LTCI certification in order to use services in the future [28]. It is also reasonable to assume that care-need certification would be more severe during hospitalization for clinical reasons. Furthermore, Matsuda and colleagues suggested that while LTCI application and certification of eligibility was related to the applicants’ attributes, service use was not [29]. Taking into consideration the findings of these previous studies together with the actual situation regarding LTCI service use, the point of initial service use might more accurately represent the time when care is actually needed.

Another point of consideration is that, according to Kato and colleagues, the effects of home care service use vary according to the level of care need [16]. A further study suggested that, in Japan, long-term care users who are higher than care level 3 are more likely to enter residential care than those in care levels 1 or 2 [30]. Thus, separate analyses of the low- and high-care need groups is necessary to get a clearer understanding of LTCI service use.

The purpose of this study was, therefore, to examine the effect of the use of short-stay services on duration to first admission to care facilities (herein referred to as residential care admission) for elderly persons certified as eligible for long-term care insurance in 2 basic care need categories, (1) low-care need group and (2) high-care need group. If indeed the use of short-stay service delays the duration to residential care admission, as we hypothesize, it will help to fulfill the wishes of many elderly people who want to stay at home for as long as possible. Importantly, it may also help to reduce increasing government expenditures on long-term care, and also offer support for family caregivers.

## Materials and methods

In this study, anonymous LTCI claims data from Ibaraki Prefecture was analyzed with permission of the Ministry of Health, Labour and Welfare (MHLW) of Japan. In 2015, the percentage of people living in Ibaraki aged 65 years or over was 26.5% (about 770,000 people), which is proportionally the same as the national figures for Japan. The proportion of individuals certified as being eligible for LTCI benefits among those aged 65 and over was 15.0%, which is a little lower than that of Japan as a whole (18.1%). The LTCI claims data, used in our analysis includes the LTCI service users’ sex, month and year of birth, start and end date of eligibility, eligibility level, long-term care services code, and month and year of when long-term care services were provided. However, these data do not include information on caregivers.

The participants of the present study were individuals who had, for the first time, been certified eligible for long-term care insurance and had subsequently moved into LTCI facilities, between April 2006 and March 2012 (n = 7,124). Participants were excluded if they were under 65 years old when they began using services (n = 1,280) (the eligibility criteria of LTCI differs for those under 65 and thus this category cannot be used for comparison) or did not meet the care-need levels at the initial use of care services (n = 303) because they were not eligible for nursing home admission. In order to standardize the baseline characteristics, participants who moved to residential care at the time of their initial use of care services (n = 1,559) were also excluded. To ensure that service use occurred before admission, participants who moved into residential care during the same month of their initial use of care services (n = 1,528) were also excluded. Finally, data of the remaining 2,454 participants were analyzed.

Participants were divided into 2 groups based on their care-need level at the initial services use: the low-care need group comprised those of care levels 1 and 2, and the high-care need group comprised those of levels 3, 4, and 5.

The measured outcome of our study was the number of months from the initial use of services until admission to LTCI facilities, including nursing homes, health service facilities for the elderly, and sanatorium-type medical care facilities. The main independent variable was the usage of short-stay services before admission.

### Statistical analysis

The interval between initial use of services and first residential care admission was tested using the log-rank test (Kaplan-Meier method). Using Cox models, we then estimated the effect of short-stay service use on the duration between the initial use of care services to first residential care admission by the level of care need. We adjusted for age at the time of initial use of care services, gender, the use of each home service, home-help, home-visit bathing, home-visit rehabilitation, home-visit nurse, day care, day rehabilitation, and rental services for assistive devices.

Analyses were done using Stata 13.1 and IBM SPSS Statistics Version 22.0 (IBM Japan, Ltd). The use of these data in this study has been officially approved to use the secondary data from Statistics and Information Department of the MHLW. Ethical approval for the study was obtained from the Ethical Committee of the University of Tsukuba, Japan (Approval No. 1009).

## Results

Table 1 shows the characteristics of each care need group. Of the 2,454 participants, 1,338 were categorized into the low-care need group, and 1,116 were categorized into the high-care need group. In the low-care need group, 915 (65.4%) were women; the mean age (years) ± standard deviation (SD) at initial use of services was 82.1 ± 7.17; and 857 (64.1%) used short-stay services. In the high-care need group, 771 (69.1%) were women; the mean age (years) ± SD at initial use of care services was 81.6 ± 8.33; and 854 (76.5%) used short-stay services.

**Table 1:** Characteristics of elderly by care need group.

		Care need group			
		low (n = 1338)		high (n = 1116)	
		n	%	n	%
Sex	Male	423	31.6%	345	30.9%
	Female	915	65.4%	771	69.1%
Age^a^	65–74	188	14.0%	233	20.9%
	75–84	611	45.7%	420	37.6%
	≧85	539	40.3%	463	41.5%
Care need level^a^	Care level 1	729	54.5%	–	
	Care level 2	609	45.5%	–	
	Care level 3	–		567	50.8%
	Care level 4	–		374	33.5%
	Care level 5	–		175	15.7%
Short-stay service	use	857	64.1%	854	76.5%
	non-use	481	35.9%	262	23.5%
Each home service use or not					
Home-help	Use	447	33.4%	284	25.4%
Home-visit nurse	Use	109	8.10%	128	11.5%
Home-visit rehabilitation	Use	17	1.30%	22	2.0%
Home-visit bathing	Use	40	3.00%	69	6.2%
Day care	Use	807	60.3%	488	43.7%
Day rehabilitation	Use	328	24.5%	234	21.0%
Rental services for assistive devices	Use	546	40.8%	594	53.2%

Data source: Long-term care insurance claims data

^a^ Data from time of initial use of any care service after certification of eligibility for long-term care insurance.

**Fig 1:**
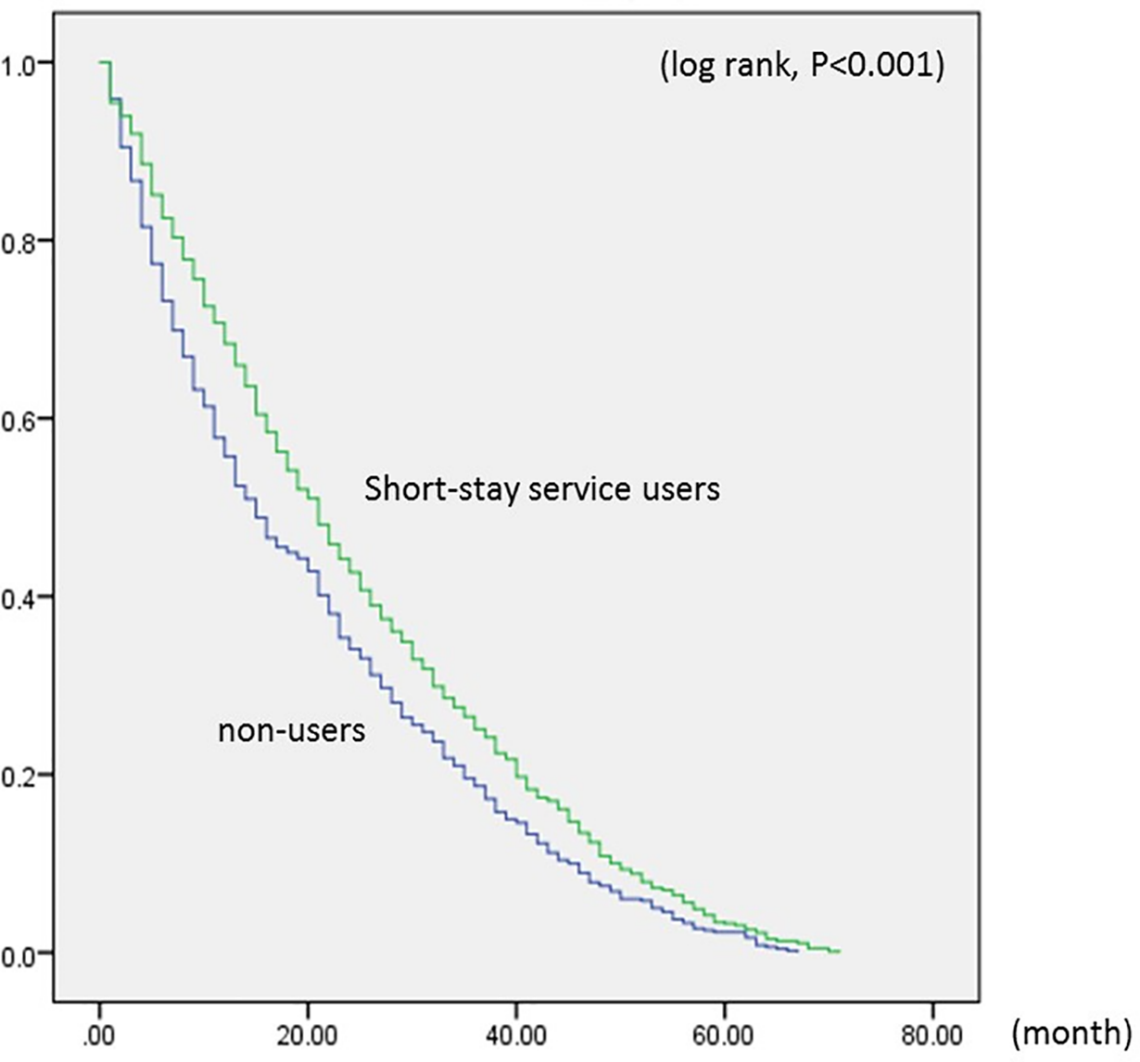
Kaplan-Meier curves showing the number of months in the low-care need group. These Kaplan-Meier curves show the comparison of the number of months from initial use of services until residential care admission between short-stay service users and non-users in the low-care need group. Data source: Long-term care insurance claims data.

**Fig 2:**
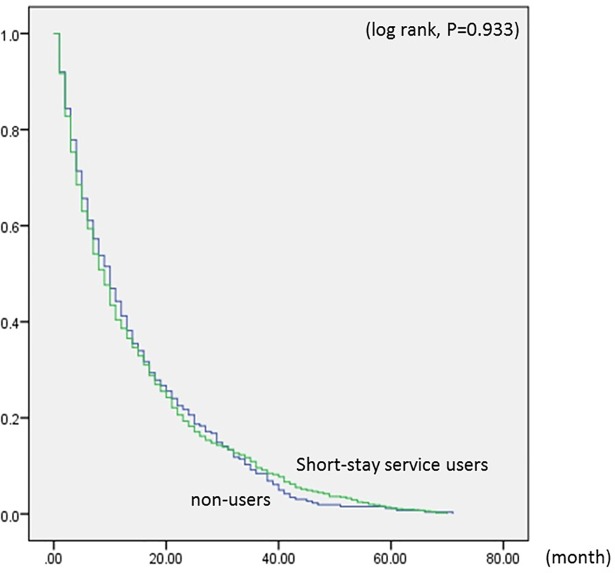
Kaplan-Meier curves showing the number of months in the high-care need group. These Kaplan-Meier curves show the comparison of the number of months from initial use of services until residential care admission between short-stay service users and non-users in the high-care need group. Data source: Long-term care insurance claims data.

Short-stay service users in the low-care need group had a delayed time-to-residential care admission compared to non-users (log rank, *P* < .001) (Fig 1). For the low-care need group, the average duration between initial use of care services to admission was 24 months for short-stay users and 20 months for non-users. In the high-care need group, on the other hand, Kaplan-Meier curves were similar between short-stay service users and non-users (log rank, *P* = .933) (Fig 2), with the average duration for both being 14 months.

Tables 2 and 3 show the results of the regression models for each care need group. In the low-care need group, even after adjusting for possible confounding factors, short-stay service users were 0.834 (95% CI: 0.740–0.939) times less likely to be admitted to residential care than non-users. However, in the high-care need group, short-stay service users were 1.254 (1.084–1.451) times more likely to be admitted to residential care.

**Table 2:** Adjusted hazard ratios (HR) and 95% confidence interval (CI) for residential care admission in the low-care need level group.

	Adjusted HR	95%CI			
Use of short-stay services	0.834	0.740	-	0.939	*
Women (ref.;men)	0.815	0.724	-	0.917	*
Age^a^ 65–74	1				
75–84	0.893	0.756	-	1.054	
≧85	0.971	0.818	-	1.153	
Use of home-help	0.820	0.728	-	0.923	*
Use of home-visit bathing	0.926	0.667	-	1.284	
Use of home-visit nurse	0.981	0.799	-	1.205	
Use of home-visit rehabilitation	0.879	0.539		1.433	
Use of day care	0.808	0.715	-	0.913	*
Use of day rehabilitation	0.834	0.728	-	0.954	*
Use of rental services for assistive devices	0.837	0.745	-	0.941	*

Data source: Long-term care insurance claims data

The outcome variable was the number of months from the initial use of services until first residential care admission

^a^ Data from time of initial use of any care service after certification of eligibility for long-term care insurance.

*P < .05

**Table 3:** Adjusted hazard ratios (HR) and 95% confidence interval (CI) for residential care admission in the high-care need level group.

	Adjusted HR	95%CI			
Use of short-stay services	1.254	1.084	-	1.451	*
Women (ref.;men)	0.980	0.859	-	1.119	
Age^a^ 65–74	1				
75–84	0.970	0.824	-	1.142	
≧85	1.083	0.918	-	1.278	
Use of home-help	0.853	0.739	-	0.984	*
Use of home-visit bathing	0.772	0.599	-	0.995	*
Use of home-visit nurse	0.790	0.649	-	0.962	*
Use of home-visit rehabilitation	1.034	0.669		1.596	
Use of day care	0.566	0.496	-	0.647	*
Use of day rehabilitation	0.636	0.541	-	0.749	*
Use of rental services for assistive devices	0.783	0.682	-	0.899	*

Data source: Long-term care insurance claims data

The outcome variable was the number of months from the initial use of services until first residential care admission

^a^ Data from time of initial use of any care service after certification of eligibility for long-term care insurance.

*P < .05

## Discussion

Our investigation has two main findings that both suggest the importance of appropriate timing of short-stay services: first, use of short-stay services was associated with delay of residential care admission in the low-care need group; and second, it was associated with earlier admission in the high-care need group after adjusting potential confounders. The first of these findings is consistent with the conclusions of Tomita and colleagues, who reported that respite care is effective in preventing hospitalization and institutionalization [25]. Our study, which measures from the time of initial service use, is arguably more accurate than previous studies in its representation of the time when care is actually needed. Our results also suggest that during the period between initial use of services to admission, the use of short-stay services in the low-care need group results in a greater deterioration of the user’s care need level (75.6%) than is observed in non-users over the same period (66.1%) (data not shown), a finding that is consistent with that of Kato and colleagues (2009) [16]. The reason why short-stay services might delay residential care admission while deteriorating the care need level could be because such services are being used at the discretion of family as a form of respite care. One limitation of the current study is the lack of information on the participants’ caregivers (caregiver information is not part of LTCI claims data). Kuchimura reports that, in many cases, the use of short-stay service occurs more often in the interests of the caregiver rather than the interests of the recipients [31]. The caregivers’ condition was a more relevant factor to home discharge than patients’ physical function [32]. Some studies have shown that the burden of caregiving is reduced by using respite care [23,24] and that the alleviation of caregiver burden is essential to prevent or delay residential care admission [33]. Moreover, Kuzuya and colleagues suggested that, while the reduction of caregiver burden and improvement of caregiver well-being may prevent the deterioration of caregiver health, it could also reduce adverse health outcomes for care recipients [23]. According to the findings of a study by Kuchimura, effects of short-stay service are better when the recipients are in agreement to use such services [31].

The second finding of our study, that using short-stay services correlated to earlier admission to residential care in the high-care need group, is similar to the findings of Ishizuki and colleagues that elderly in residential care had used nursing home short-stay services more than long term dwellers [26]. This could be due to the fact that users of short-stay services in the high-care need group have a greater desire to move into residential care than non-users and, because of a paucity of nursing homes in Japan, are using short-stay services while they are waiting for nursing home vacancies. Meanwhile, non-users in the high-care need group (23.5%, see Table 1) may be those who are determined to stay at home and refuse to stay at a nursing home facility even for a few days of short-stay service.

Besides short-stay services, we adjusted for additional variables that could possibly relate to stay-at home duration within both care need groups. We found that home-help, day care, day rehabilitation, and rental services for assistive devices, are related to stay-at home duration in both care need groups, while the use of home-visit bathing and home-visit nursing were correlated in the high-care need group alone. Although such services were related to delay to admission to residential care, only the use of short-stay services showed opposing results in the low- and high-care need groups suggesting that timing of short-stay service use is particularly important.

From April 2015, as a Japanese government initiative to promote care at home, a new law states that elderly with a low-care need level (care level 1 or 2) cannot be put into nursing homes [34]. Taken together, our findings suggest that in order to extend the stay-at-home duration for the elderly, it is necessary to make use of short-stay services at appropriate times, especially for those within the low-care need levels. The use of such services benefits not only care recipients, but also caregivers. Kuzuya and colleagues suggested that adverse outcomes in care recipients whose caregivers carry the highest level of care burden are more evident in non-users of respite services than in service users [23]. Early use of respite services is, therefore, necessary for the care caregiver in order to prevent excessive stress while maintaining their own quality of life and achieving self-actualization.

There were a number of limitations to our study. We used anonymous LTCI claims data that did not include the participants' medical conditions or their income level, which may have some implications. The presence of a disease and its severity might effect stay-at-home duration [25]. Income level might be associated with admission because formal services use is higher for upper-income households than others [10]. Another limitation was that we could not adjust for the situation of informal care to the elderly as this data was not available. A recent study by Salminen and colleagues suggests that informal care is the key determinant of whether or not an elderly person is institutionalized [35]. Caregiver characteristics and caregiving situation have also been shown to relate to informal caregiver attitudes towards respite care [36]. Therefore, the presence or absence of family caregivers and the situation of informal care should be included in future studies. However, we used LTCI claims data despite lacking information of caregivers because it would be sufficient to clarify this issue, to test our hypothesis, the usage of short-stay services would lead to an increase in the duration of stay-at-home. The final limitation of our study is that we did not take into consideration the number of days used for short-stay services and it could be that frequent users of the service are more likely to move into residential care than infrequent users.

Taken together, our results suggest that, in light of the increasing number of disabled elderly and limited resources, it is necessary to use appropriate services at appropriate times in order to delay or prevent residential care admission. By doing so we can take positive steps towards fulfilling the wishes of those elderly who want to stay at home for as long as possible, reducing the burden of the caregivers, and using services in cost-effective manner.
